# Voyages in Vacation.—XII

**Published:** 1888-12-15

**Authors:** 


### Voyages in Vacation.—XII.

By One in Search of Restful Restoratives.

(*Continued from p.* 151.)

### RUSSIAN PALACES AND CHURCHES.

Magnificent and wonderful as the Winter Palace is in many respects, that of the Tsarskoé Selo surpasses it altogether. This palace is situated about 25 versts from St. Petersburg, and is easily reached by railway. It forms the favourite summer residence of the Imperial Court; Peter I. having built a cottage and hothouse here, to which he added a zoological garden. It first became an Imperial residence in the time of Katherine I. and Elizabeth, but to Katherine II. belongs the credit of its present beauties and magnificence. We have not space to describe the grounds which surround the palace, and are eighteen miles in circumference, or the many extraordinary buildings, summer houses, dolls' houses of the young Grand Duchesses, where they carry on a mimic ménage, the Chinese tower, the theatre, the Chinese village, the Dutch and Swiss cowhouse, the Turkish kiosk, the marble bridge with Corinthian columns of polished marble, the granite pyramid, fields of roses, hermitages, artificial ruins, Roman tombs, grottoes, and waterfalls. On the lake close to the palace are a fleet of pigmy vessels, with a collection of the boats and canoes of all nations, including the gilt barges of Katherine II. and a popofka, or circular monitor. All these, and much more, must be seen to be realised and appreciated. This palace and its history give some idea of the reckless expenditure lavished upon royal residences in this imperial land. Thus, Katherine II. is said to have covered with gold-leaf every statue, pedestal, and capitol of the numerous columns as well as all the vases, carvings, and ornaments. The cost is said to have been quite half a-million sterling, and when some of it wore off, the contractors are stated to have offered Katherine a quarter of a-million for the fragments of gold-leaf that were left.

Some of the rooms in this sumptuous palace are so magnificent as to pass belief. Thus, there is a lapis lazuli room, the floor of which is ebony inlaid with mother-of-pearl, the effect produced being indescribable. Then there is an amber room, another marvellous place, the walls of which consist entirely of amber in all sorts of designs. Amber frescoes project from the walls forming groups of figures, and the appearance of this apartment is dazzling in its grandeur. The walls and floors throughout the palace are sumptuously decorated, the walls in white and gold—hung with rich silk curtains, and the floors covered with the most resplendent designs of parquet, the colours and arrangement of which leave nothing to be desired.

Then there is one remarkable room, situated in the grounds, where Emperor and Empress can dine entirely alone. The tables are so arranged that on touching a spring they descend to the apartment below, where such viands as the Royal personages may have ordered on the slate menu are placed upon the boards which, when loaded, ascend to their places. In this way entire solitude is secured, no waiters or attendants of any kind ever enter the apartment, and the guests can disport themselves at their own sweet will and pleasure without interruption or inspection.

Lastly, there is the banqueting hall, the walls of which for the greater part are covered entirely with gold. Indeed it may be said that the whoie of the ceilings of the principal apartments are covered with this metal. All attempts at description must fail to convey to the reader any adequate idea of the resplendent beauties and surprising lavishness of this wonderful palace. Full of historical interest, beautifully situated, and containing all the many wondrous things which we here briefly note, there can be no doubt that the Tsarskoé Selo is a palace with few rivals, and, possibly, without a peer.

We have already alluded to the shrines to be found almost everywhere in the streets and places of public resort in every town throughout Russia. Besides these, one soon comes to realise that every room, or almost every room, in the Russian houses, and also the shops and hotels, have shrines or the holy *ikon* (a small painting usually representing the patron saint) suspended in the corner of every room, for which reason a Russian invariably takes off his hat whenever he enters an apartment however humble, or even a shop. The amount of crossing, genuflexion, and signs of various kinds in which the natives indulge before shrines, and in the churches, has to be witnessed to be believed. There are no seats in the chuches, the congregation stand or kneel, and during the whole of the service they bow and cross themselves with each movement, whilst the more devout prostrate themselves on the stone floor, which they often kiss. It is no uncommon sight to witness a mother teaching her children to so prostrate themselves, and to touch the flags with their foreheads, each time the action is repeated. As in most Roman Catholic churches abroad, people pass in and out, and move about here and there throughout the whole time the service is proceeding. There are two notable features about the congregations. They consist, for the greater part, of men, and preaching does not apparently form a portion of the service, for we saw, at any rate, no pulpits in Russian churches. In most English churches women very largely predominate, but then there is always a sermon, and this may have much to do with the relative proportion of the two sexes represented by the congregations to be met with here and in Russia. When the service commences, the choir of boys standing before the closed doors of the sanctuary, which are of beautiful design and most elaborate workmanship, continuously chant their *Kyrie Eleison, Amen.* The voices are sweet in tone, but the refrain sounds monotonous to the ears of a stranger. After a considerable time the golden screen or gates, *i.e.*, the *Iconostas*, which hides the sanctuary from view, are flung open, and a magnificent stained glass window is exposed, representing the Ascension, the figure of the Saviour being of immense size. The priests, who usually have long flowing beards and hair, pass from the sanctuary through the open gates, chanting in deep and impressive tones, and so the service proper commences. This description represents the ceremony witnessed at St. Izak's Church, where the interior is most magnificent, the columns on either side of the *Iconostas* being covered with malachite and lapis lazuli. Many of the more ancient and esteemed pictures, or *ikons*, as they are called, are covered with precious stones, those on *The Ikon of the Virgin* in the Kazan Cathedral being valued at £15,000. It is a remarkable fact that, whereas the Russian Church condemns idolatry, and so rejects all massive images of the Saviour and others as idolatrous, it permits and indeed encourages pictures, mosaics, basreliefs and anything of the kind which can be represented on a flat surface, the cathedrals and churches being literally filled with oil paintings, the new church of "Our Saviour" at Moscow, probably the finest Russian church in existence, being especially noteworthy for the artistic excellence of its pictures.

The first thing a Russian does on entering a church is to purchase from an attendant one or more wax candles, the sale of which produces a large sum. Taking the candle in his hand, he proceeds to the shrine before which he desires to offer his prayers, crossing himself and kneeling at intervals as he slowly approaches, and, having lighted his candle at the holy lamp and fixed it in the stand provided for the purpose near the shrine, he falls on his knees. Having finished his orisons, he rises slowly and retires backwards from the shrine, bowing and crossing himself as before, until he has placed a considerable distance between himself and it. In addition to the St. Izak's and Kazan Cathedrals at St. Petersburg, no one should fail to visit that of St. Peter and St. Paul, in the fortress where all the sovereigns of Russia since the foundation of St. Petersburg lie buried, except Peter II. All these cathedrals are disappointing interiorly owing to the gloom which is present almost constantly. Everywhere this darkness prevents the many beautiful architectural features from being properly seen, and creates a heavy appearance, so that, except at Easter, or upon some great festival when the interior is brilliantly illuminated the effect is almost entirely lost. The lighting at the Church of Our Saviour, at Moscow, leaves however little if anything to be desired. There is a very fine view of St. Petersburg and the country for miles around from the dome of St. Izak's Cathedral, which all who are not afraid of many steps (300 ft.), should make a point of seeing on a really fine day. We ought, perhaps, to add before leaving this subject, that no women are ever admitted into the inmost shrine or sanctuary of a Russian church.

(*To be continued.*)

